# Topological domains/domain walls and broken symmetries in multiferroics

**DOI:** 10.1093/nsr/nwz015

**Published:** 2019-01-25

**Authors:** Sang-Wook Cheong

**Affiliations:** Rutgers Center for Emergent Materials, Rutgers University, USA

The magnetoelectric domains/domain walls in intrinsic multiferroics can exhibit intriguing topological nanoscale textures. These thermodynamically stabilized nano-textures are compared with nanoscale polar structures in nanostructured composite films. In addition, we show that the consideration of broken symmetries is essential to understand the macroscopic origin of multiferroicity and also to predict new multiferroicity.

**Figure 1.**
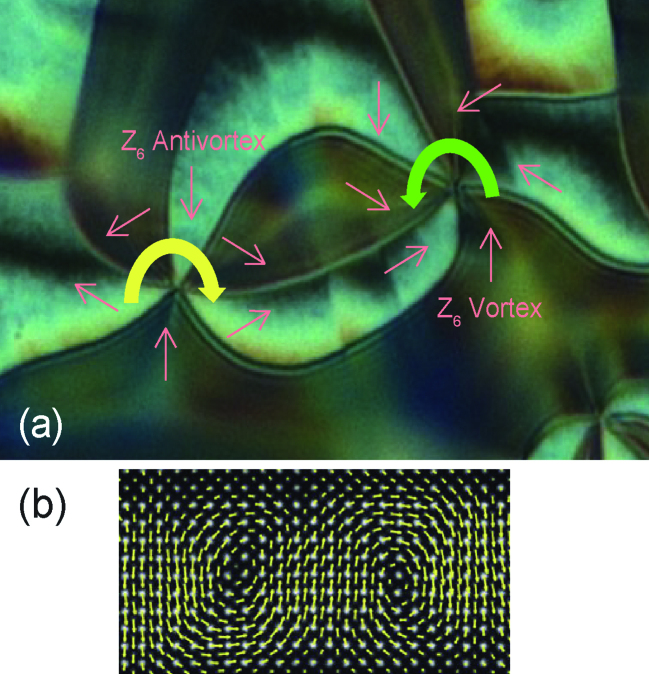
(a) Vortex–antivortex pair in the 2D-cut view of a 3D Z_6_ vortex line in hexagonal RMnO_3_. Red arrows show characteristic oxygen distortion directions in domains. From Ref. [1]. These 3D Z_6_ structural vortex lines become 3D Z_6_ magnetic vortex lines when spins order below ∼80 K. (b) Nanoscale polar screws in PbTiO_3_/SrTiO_3_ superlattice films. From Ref. [3]. Mirror as well as inversion symmetries are broken in these polar screws.

Multiferroics, where ferroelectric and magnetic orders coexist, has attracted an enormous amount of attention in recent years because of the cross-coupling between magnetism and ferroelectricity, and the related possibility of controlling magnetism with an electric field (and vice versa). These multiferroics with various broken symmetries always accompany domains and domain walls. Thus, the study of the magnetoelectric properties of these domains and domain walls as well as the spatial configurations of these domains and domain walls are quintessential to understanding and utilizing bulk magnetoelectric effects in multiferroics. One important discovery in multiferroic domains/domain walls is 3D Z_6_ vortex lines, resulting in a topological vortex–antivortex structure, in a 2D-cut view of hexagonal RMnO_3_ (R = rare earths) (see Fig. 1a) [1]. 2D oxygen distortion vectors rotate around the vortex core, and rotate around the antivortex core in the opposite manner. Similar 3D Z_8_ vortex lines were found in Ca_2_SrTi_2_O_7_, which is closely related to so-called hybrid improper ferroelectrics such as Ca_3_Ti_2_O_7_ [2], where the simultaneous presence of two-different oxygen cage distortions induces polarization. It turns out that these 3D vortex lines are rather common, as long as long-range interaction such as strain or dipolar coupling is not dominant. Intriguing nanoscale polarization textures such as nanoscale polar screws in PbTiO_3_/SrTiO_3_ superlattice films have also been discovered [3]. These polar screws accompany broken space-inversion and mirror symmetries. These polar screws are distinct from ferro-rotation (sometime called ferro-axial or 2D chiral) where space inversion is not broken [4]. It should be emphasized that topological vortex lines form through a thermodynamic process such as a thermal phase transition, but nanoscale polarization textures such as polar screws result mostly from constraints or boundary conditions. For example, the size and density of topological vortex domains depend on the cooling rate across a phase transition, but nanoscale polarization textures such as polar screws can be realized only in nanostructured films.

**Figure 2.**
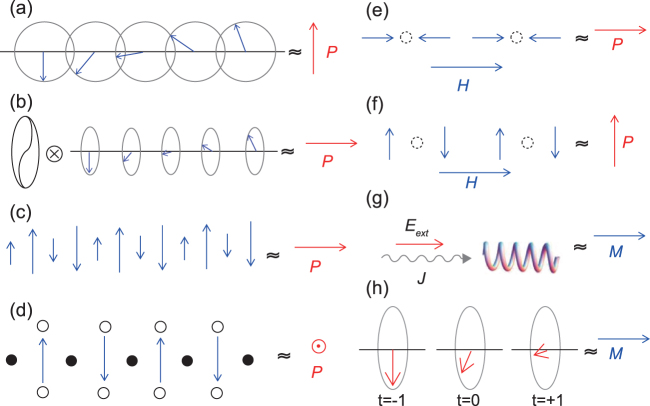
Various (quasi-)1D entities having an SOS relationship with polarization (*P* ) or magnetization (*M*). Blue arrows are spins, and red arrows are polarizations. (a) Cycloidal spin order. (b) Ferro-rotational lattice with helical spin order. (c) Two-different types of spins with up–up–down–down spin order. (d) Simple antiferromagnetic order with oxygens below (solid circles) and above (open circles) the paper plane. (e, f) Simple antiferromagnetic order with alternating in-chain oxygens (dashed circles) in a magnetic field (*H* ). (g) Current flow with external electric field in a screw-like chiral lattice. (h) Polarization rotating with time.

It turns out that intrinsic multiferroicity can often be understood from the consideration of broken symmetries. Magnetic order naturally breaks time-reversal symmetry, and a spatially varying magnetic lattice, combined with a centrosymmetric crystallographic lattice above a magnetic transition temperature, can break space-inversion symmetry, leading to multiferroicity through the spin–orbit interaction, called magnetism-driven ferroelectricity (MDF). In these MDF compounds, magnetism and polarization (*P*) are strongly coupled, so, for instance, polarization or dielectric constant changes drastically when an applied magnetic field (*H*) can induce a magnetic phase transition. This phase-transition-driven magnetoelectric effect can be termed the ‘colossal magnetoelectric effect’, similar to the colossal magnetoresistance effect in ferromagnetic perovskite manganites where an applied magnetic field induces a metal–insulator transition, so electric resistance changes drastically. A large number of new MDF compounds have been discovered during the last decade. Since polarization (and magnetization (*M*)) is a 1D entity (in fact, a (pseudo-)vector), (quasi-)1D schematics with broken symmetries can often explain the multiferroicity in most MDF materials [4]. We will use the following notation for the various symmetry operations: **R** = π rotation operation with the rotation axis perpendicular to the *P*/*M* direction, **R** = π rotation operation with the rotation axis along the *P*/*M* direction, **I** = space inversion, **M** = mirror operation with the mirror plan perpendicular to the *P*/*M* direction, **M** = mirror operation with the mirror plane containing the *P*/*M* direction, **T** = time-reversal operation. For example, {**R**, **I**, **M**} is the set of all broken symmetries of *P*, and all (quasi-)1D spin configurations on the left-hand side of Fig. 2a–d also have broken {**R**, **I**, **M**}. Thus, they do have a symmetry operational similarity (SOS, shown by the ‘≈’ symbol in Fig. 2) with polarization. Note that since we consider the 1D nature of *P*, translational symmetry along the 1D direction is irrelevant, and additional broken symmetries in the left-hand-side spin configurations, such as **R** (different from π or 2π rotation) in Fig. 2a, are also not relevant. The cycloidal-spin-order-driven multiferroicity in e.g. TbMnO_3_ and LiCu_2_O_2_ corresponds to Fig. 1a [5,6], and the multiferroicity driven by a ferro-rotational lattice with helical spin order in e.g. RbFe(MoO_4_)_2_ and CaMn_7_O_12_ can be explained with Fig. 2b [7,8]. Figure 2c corresponds to multiferroicity with two (or more than two) different magnetic sites in Ca_3_CoMnO_6_, TbMn_2_O_5_ and orthorhombic HoMnO_3_ [9–11]. The so-called p–d hybridization multiferroic Ba_2_CoGe_2_O_7_ can be described by Fig. 2d [12]. The left-hand-side entities in Fig. 2e and f with zero *H* only have broken {**I**, **M**}, but the left-hand-side entities with non-zero *H* have now broken all of {**R**, **I**, **M**}, so becoming SOS with *P*, which is consistent with e.g. the linear magnetoelectric (ME) effects in Cr_2_O_3_ (Fig. 2e; diagonal linear ME for low *H*, and Fig. 2f; off-diagonal linear ME for large *H* beyond the spin–flop transition *H*) [13]. It is also interesting to consider how to induce magnetization in non-magnetic materials in a non-trivial manner. {**R**, **M**, **T**} is the set of all broken symmetries of  *M*. Since any static configuration of *P* cannot break **T**, a time component has to be incorporated into the *P* configuration. Two examples having SOS with *M* are shown in Fig. 2g and h. When electric current is applied to a tellurium crystal with a screw-like chiral lattice, corresponding to Fig. 2g, *M* can be induced [14]. Figure 2h can be realized when a Néel-type ferroelectric wall moves in the direction perpendicular to the wall. This situation has not been experimentally realized, but the dynamics of multiferroicity discussed in Ref. [15] is relevant to this case. We emphasize that the above symmetry arguments reveal neither the microscopic mechanism for multiferroicity nor the magnitude of the induced *P* or *M*. However, it turns out that e.g. all magnets with cycloidal spin order, corresponding to Fig. 2a, always show experimentally observable *P*. Thus, these situations appear to resonate with the famous statement of Murray Gell-Mann: ‘Everything not forbidden is compulsory.’ In other words, when multiferroicity is allowed by the consideration of broken symmetries, the effect is often large enough, and so experimentally observable.

In summary, the intriguing topological structures of magnetoelectric domains/domain walls in multiferroics have been discovered and need to be further explored. In particular, the understanding of the magnetoelectric and dynamics properties of domain walls in multiferroics is at a primitive stage and needs to be further investigated. The broken-symmetry consideration with 1D entities is a strong starting point to understand multiferroicity. Furthermore, with the concept of SOS, it is rather straightforward to predict new spin textures and polarization textures that induce polarization and magnetization, respectively. Thus, this simple but powerful approach will open a new avenue to discover new multiferroic materials and phenomena.
